# A death in the family: Sea lamprey (*Petromyzon marinus*) avoidance of confamilial alarm cues diminishes with phylogenetic distance

**DOI:** 10.1002/ece3.3930

**Published:** 2018-03-09

**Authors:** John B. Hume, Michael Wagner

**Affiliations:** ^1^ Department of Fisheries & Wildlife Michigan State University East Lansing MI USA

**Keywords:** alarm cue, heterospecific, lamprey, predation, risk, semiochemical

## Abstract

Alarm signals released after predator attack function as reliable public information revealing areas of high risk. The utility of this information can extend beyond species boundaries, benefiting heterospecifics capable of recognizing and responding appropriately to the signal. Nonmutually exclusive hypotheses explaining the acquisition of heterospecific reactivity to cues suggest it could be conserved phylogenetically following its evolution in a common ancestor (a species‐level effect) and/or learned during periods of shared risk (a population‐level effect; e.g., shared predators). Using a laboratory‐based space‐use behavioral assay, we tested the response of sea lamprey (*Petromyzon marinus*) to the damage‐released alarm cues of five confamilial (sympatric and allopatric) species and two distantly related out‐groups: a sympatric teleost (white sucker *Catostomus commersonii*) and an allopatric agnathan (Atlantic hagfish *Myxine glutinosa*). We found that sea lamprey differed in their response to conspecific and heterospecific odors; exhibiting progressively weaker avoidance of cues derived from more phylogenetically distant confamilials regardless of current overlap in distribution. Odors from out‐groups elicited no response. These findings suggest that a damage‐released alarm cue is at least partially conserved within the Petromyzontidae and that sea lamprey perceives predator attacks directed to closely related taxa. These findings are consistent with similar observations from gastropod, amphibian and bony fish taxa, and we discuss this in an eco‐evo context to provide a plausible explanation for the acquisition and maintenance of the response in sea lamprey.

## 1 INTRODUCTION

Predation and the recognition of predation risk can be powerful selective forces, capable of shaping interactions within and among predators and prey as they seek to gain advantage in this lethal contest (Dawkins & Krebs, 1979; Genovart et al., 2010). Antipredator adaptations are diverse, including changes in gross morphology (e.g., shell thickening in mussels, Cheung, Lam, Gao, Mak, & Shin, 2004), behavioral plasticity (e.g., varying antipredator behaviors depending on predator–prey syntopy in mosquito larvae, Roux, Diabaté, & Simard, 2013), and alterations in life‐history strategy (e.g., production of diapausing eggs and males in *Daphnia* spp., Ślusarczyk, 1999), each of which is costly to produce (Taylor, Balph, & Balph, 1990). One set of conditions that can lead to the evolution of such defenses stems from the threat of predation, if it can be accurately assessed and individuals subsequently avoid capture (Lima & Dill, 1990; Sih, 1985). One such tactic, predator detection warnings, can be relatively fine‐tuned. For example, certain primates produce distinct alarm calls in response to aerial versus terrestrial threats, allowing for application of the most effective antipredator tactic (Seyfarth, Cheney, & Marler, 1980). Recent studies have revealed predator recognition and subsequent alarm calls in birds to be even more nuanced, incorporating information relating to predator size (Templeton, Greene, & Davis, 2005) and the distance between predator and prey (Leavesley & Magrath, 2005).

Aquatic animals are also under strong selection pressure to accurately assess predation risk in a variety of contexts and evolve suitable morphological, behavioral, and life‐history traits (Brown, Rive, Ferrari, & Chivers, 2006; Chivers, Zhao, Brown, Marchant, & Ferrari, 2008; Ferrari, Messier, & Chivers, 2008; Helfman, 1989; Kepel & Scrosati, 2004; McCarthy & Fisher, 2000). However, in water, public information regarding predation risk often takes the form of damage‐released alarm cues; chemicals involuntarily leaked into the environment following injury that elicit antipredator responses in conspecifics (Acquistapace, Calamai, Hazlett, & Gherardi, 2005; Barreto et al., 2013; Chivers & Smith, 1998; Ferrari, Elvidge, Jackson, Chivers, & Brown, 2010; Kicklighter, Germann, Kamio, & Derby, 2007; Mathuru et al., 2012; Smith, 1992). Alarm cues are expressed by major aquatic taxa from both freshwater and marine environments, including echinoderms (Hagen, Anderson, & Stabell, 2002; Majer, Trigo, & Duarte, 2009), mollusks (Daleo et al., 2012; Dalesman, Rundle, & Cotton, 2007; Wood, Pennoyer, & Derby, 2008), crustaceans (Hazlett, 2011), arachnids (Persons, Walker, Rypstra, & Marshall, 2001), acarids (Grostal & Dicke, 1999), insects (Gall & Brodie, 2009; Llandres, Gonzálvez, & Rodríguez‐Gironés, 2013), and fishes (Brown, Ferrari, & Chivers, 2011). Damage‐released alarm cues also mediate learned predator recognition, including the relative level of risk posed by individual predator species (Brown & Chivers, 2005; Chivers & Smith, 1998; Gherardi, Mavuti, Pacini, Tricarico, & Harper, 2011; Wudkevich, Wisenden, Chivers, & Smith, 1996), changes in the relative threat through ontogeny (Johnston, Molis, & Scrosati, 2012), and the labeling of risky habitats (Chivers & Smith, 1998).

If the threat of predation is shared across taxonomic boundaries, and heterospecifics are capable of “eavesdropping,” a selection advantage is conferred to those species capable of recognizing and responding to the risk communications of sympatric species (Magrath, Haff, McLachlan, & Igic, 2015). Several aquatic taxa avoid heterospecific alarm cues, often exhibiting a decline in the intensity of antipredator behavior with increasing phylogenetic distance between the individual attacked and the individual perceiving that event (Dalesman et al., 2007; Hazlett & McLay, 2005; Mitchell, Cowman, & McCormick, 2012; Schoeppner & Relyea, 2009). One explanation for this pattern is that individuals innately recognize the damage‐released alarm cues of closely related taxa because they were present in a common ancestor (i.e., are chemically similar). The most parsimonious view of the evolution of this phenomenon is that alarm cues initially evolved to provide separate fitness‐enhancing functions (e.g., immunity, Chivers, Wisenden et al., 2007; Chivers, Zhao, & Ferrari, 2007) and had no association with predation events specifically. However, during an attack, these and other chemical compounds may be perceived together as a mixture that is capable of providing both an indication of predation and the identity of the species involved (Faulkner et al., 2017; Wisenden, 2015). Presumably, over evolutionary time, gradual changes to these chemical compounds responsible for initiating antipredator behaviors result in only partial recognition of the cue, regardless of whether both species continue to overlap spatially and share predators. This explanation has been previously referred to as the “phylogenetic‐relatedness hypothesis” (Schoeppner & Relyea, 2009). Alternatively, the pattern may be related to shared ecology. If two distantly related yet sympatric taxa share certain predators, then one may learn to recognize the alarm cue of the other—regardless of how long ago they shared a common ancestor—if they can associate that cue with a predation event. This socially acquired predator avoidance ability is widespread among vertebrates (Griffin, 2004) and has been referred to as the “ecological‐coexistence hypothesis” (Schoeppner & Relyea, 2009).

Lampreys and hagfishes represent the oldest extant members of the vertebrate lineage, with a divergence time from gnathostomes estimated at 615 million years before present (MY, range = 391–550, Blair & Hedges, 2005; dos Reis et al., 2015; Hedges, 2001; Kuraku & Kuratani, 2006). Due in part to their comparatively simplistic vertebrate anatomy and physiology, lampreys—and sea lamprey (*Petromyzon marinus*) in particular—have become established model organisms in a variety of scientific disciplines (Docker et al., 2015; Green & Bronner, 2014). One aspect of sea lamprey biology that has garnered substantial interest is the means by which chemical cues and pheromones operate to complete the multistaged life cycle (Buchinger, Siefkes, Zielinski, Brant, & Li, 2015). Three such systems are known. First, subadults migrate into rivers to locate suitable larval rearing habitat before spawning, attracted to odors emitted from larvae as a byproduct of their feeding (Bjerselius et al., 2000; Fine & Sorensen, 2010; Meckley, Wagner, & Luehring, 2012; Wagner, Twohey, & Fine, 2009). Second, because movements during this period are restricted to hours of darkness, predator detection via vision is negated. However, lampreys are still successfully attacked by a range of generalist predators during the spawning migration (Cochran, 1986, 2009; Roffe & Mate, 1984; Sepulveda, Rutz, Ivey, Dunker, & Gross, 2013; Sjöberg, 1985, 1989). The presence of a damage‐released alarm cue and associated antipredator behavior has been recognized in sea lamprey (Imre, Di Rocco, Belanger, Brown, & Johnson, 2014; Wagner, Stroud, & Meckley, 2011). Sea lamprey reduce exposure to predation risk by spatially avoiding the source of the signal (when associated with an area of the stream) or by accelerating its upstream movement to reduce the time in the risky area (Bals & Wagner, 2012; Hume et al., 2015; Luhring et al., 2016). Finally, sexually mature females are further drawn to specific spawning sites by the presence of a sex pheromone emitted by mature males (Siefkes, Scott, Zielinski, Yun, & Li, 2003).

Despite a higher fitness cost to hybridization as phylogenetic distance increases (Piavis et al., 1970), many lampreys share nests during reproduction (Hume, Adams, Mable, & Bean, 2013 and references therein). Although reproductive opportunity is not shared across lamprey species, there is substantial overlap between them in the habitat requirements of both larvae (Dawson, Quintella, Almeida, Treble, & Jolley, 2015) and adults (Johnson, Buchinger, & Li, 2015). Lampreys should, therefore, exploit publically available information relating to habitat quality regardless of the originating species. Indeed, sea lamprey are attracted to both heterospecific larval (Fine, Vrieze, & Sorensen, 2004) and adult odors (Buchinger et al., 2016) during their reproductive migration, because the cues represent honest signals of rearing and spawning habitat quality that are less costly to pursue compared with direct evaluation of stream habitats (Wagner et al., 2009). Additionally, cross‐reactivity to damage‐released compounds from two lamprey species (silver lamprey *Ichthyomyzon unicuspis*; Bals & Wagner, 2012; and Pacific lamprey *Entosphenus tridentatus*; Byford, Wagner, Hume, & Moser, 2016) has been reported, suggesting this may also be a trait shared by all northern hemisphere lampreys (Petromyzontidae) that would enable the avoidance of predators currently attacking any lamprey species.

The relationship between phylogenetic relatedness and response to heterospecific alarm cues is unknown in lampreys. However, the observed sea lamprey attraction response to larval odors was not weaker for a more distant relative (Fine et al., 2004), suggesting shared ecology (spawning and larval habitat requirements) may be sustaining the heterospecific reactivity observed in sea lamprey. Therefore, we examined the behavioral response of sea lamprey to damage‐released odors extracted from five other species of lamprey from North America and two distantly related taxa, Atlantic hagfish (*Myxine glutinosa*) and white sucker (*Catostomus commersonii*). Only sea lamprey derived from a single Laurentian Great Lakes population were used as respondents, enabling control of any effect of prior experience or learning that may occur in respondents collected from areas differing in natural predation pressure (e.g., Dalesman et al., 2007). Four of the species of lampreys tested are sympatric with sea lamprey in the Great Lakes, as are white sucker, and the additional species of lamprey and Atlantic hagfish have allopatric distributions. Consistent with the ecological‐coexistence hypothesis that a migrating lamprey should avoid all cues pertaining to lamprey‐specific attack, we predicted that sea lamprey would avoid the extracted alarm cues from all lamprey species equally, but ignore odors from the out‐groups. Alternatively, if the phylogenetic‐relatedness hypothesis applies, we expected the intensity of avoidance of extracted lamprey alarm cues would diminish with phylogenetic distance.

## 2 MATERIALS AND METHODS

### 2.1 Animal collection and alarm cue extraction

We obtained wild specimens of six species of lamprey for extracting alarm cues and a single hagfish and teleost species for use as out‐groups. All lamprey species and the teleost were captured during their annual spring spawning migrations. Sea lamprey were collected from the Cheboygan River, Michigan during May 2017. Chestnut lamprey *Ichthyomyzon castaneus* collected in the St. Joseph River, Michigan; silver lamprey *I. unicuspis* from the Peshtigo River, Wisconsin; northern brook lamprey *I. fossor* from Canada Creek, Michigan; and American brook lamprey *Lethenteron appendix* were collected from Betsie River, Michigan, all in April–May 2016. Pacific lamprey *E. tridentatus* were obtained from the lower Columbia River, Washington in April 2015. Atlantic hagfish *M. glutinosa* were obtained from Huntsman Marine Science Centre, St. Andrews, New Brunswick, Canada during summer 2013, and a single white sucker *C. commersonii* was collected in the Black Mallard River, Michigan in May 2017. All carcasses were kept frozen at −20°C prior to extraction. All extractions took place between May and June 2017, with the exception of Atlantic hagfish extracted in 2014 and Pacific lamprey extracted in April 2015. Long‐term storage (>3 years at −20°C) of damage‐released odor extracted in this manner does not reduce its efficacy in behavioral assays (J. B. Hume, unpublished data).

Because the species used in this experiment differ substantially in body size, the mass of a single male sea lamprey (255 g wet weight) carcass was used to standardize the mass of tissue extracted from all species, to ensure similarity across whole‐body extracts. Due to their larger size, only single sea and Pacific lamprey carcasses were used, whereas approximately five silver lamprey carcasses were used, eight chestnut lamprey, 130 northern brook lamprey, and 150 American brook lamprey. A single white sucker carcass was used to extract odors and approximately four Atlantic hagfish carcasses.

Odor extractions for all carcasses and the behavioral assay followed the protocol first outlined in Bals (2012) and established in the literature in Bals and Wagner (2012). Briefly, 1 L capacity 71/60 Soxhlet apparatuses (Ace Glass Inc., Vineland, New Jersey) with attached water‐cooled Allihn condensers and 1 L solvent reservoirs were heated by a hemispherical mantle to 75–80°C. One liter of solvent (50:50 weight/weight of 200‐proof ethyl alcohol and deionized water) was added to the solvent reservoir. Lastly, 255 g of tissue (either a single carcass or carcasses) was added to the extractor body. Extractions were fully cycled three times each. Extracts were then strained and held at −20°C prior to experimental use.

### 2.2 Experimental apparatus

**Figure 1:**
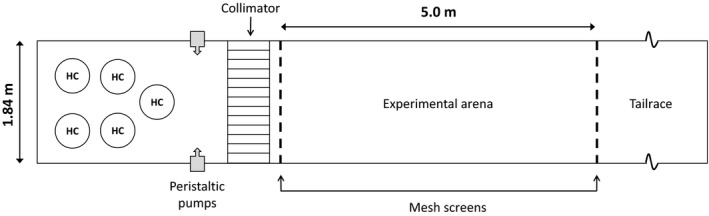
Plan view of laboratory space‐use assay. Sea lamprey were acclimated in holding cages (HC) within the upstream section (leftmost) before being moved downstream into the experimental arena. Stimulus odors were applied via one of two peristaltic pumps. The experimental arena itself was demarcated into four equal sections using a white base and contrasting tape to enable easy detection of experimental subjects on infrared‐sensitive cameras positioned overhead

Given their nocturnal habits during spawning migration, all behavioral trials using sea lamprey took place at night (2200–0300 hr) from 22 June 2017 to 30 June 2017. Trials were conducted at the U.S. Geological Survey's Hammond Bay Biological Station (HBBS, Millersburg, Michigan, USA) within two concrete raceways (20 × 1.84 m) partitioned into an upstream area for holding animals (7.5 m long), an experimental arena (5.0 m), and tailrace section (7.5 m) with mesh barrier screens (Figure 1). Immediately, upstream of each experimental arena, and downstream of the inflow, collimators were positioned to produce laminar flow and discrete odor plumes. Lake Huron water was drawn in through an offshore intake pump (temperature 12.7–15.5°C) and supplied to each raceway continuously. Discharge was maintained between 9 and 10 L/s. Although adult sea lamprey can perceive long wavelength light (Morshedian et al., 2017), infrared lamps do not alter their natural behavioral tendencies in captivity. Therefore, both experimental arenas were monitored by infrared‐sensitive video cameras (Axis Communications; Q1604 Network Camera) positioned above the center of the arena, in conjunction with two infrared lights (Wildlife Engineering; Model IRLamp6). Both camera feeds were observable from another area using video monitors, and trial footage was preserved on digital media (1 TB Western Digital, My Passport Ultra HD) for future analysis.

Stimulus odors (alarm cue and ethanol control) were applied to one side of a raceway via peristaltic pumps (MasterFlex model 7533‐20) at a rate of at 20 ml/min from the upstream end of each experimental arena, 15.2 cm from either the left or right side. Before applying extracted odors to the raceways, 8 ml of extracted odor (or ethanol control) was combined with 392 ml of Lake Huron water drawn from raceways into 500 ml Erlenmeyer flasks and stirred continuously using a 2‐cm‐long magnetic stir bar and associated stir plate (Cole‐Parmer). Combining raceway water with extracted odors resulted in a final dilution of 1 μl/L, capable of eliciting strong repellency in conspecific and heterospecific trials (Bals & Wagner, 2012). Cross‐contamination of alarm cues between species was prevented using separate pumps, tubing, glassware, and stir bars for all stimuli.

### 2.3 Experimental animals

Male sea lamprey (359–589 mm TL, mean = 485 mm; 90–432 g, mean = 246 g) were obtained by U.S. Fish and Wildlife Service staff during an annual sea lamprey monitoring program in May and June 2017. These lamprey were actively migrating to spawning grounds in two rivers flowing into Lake Huron (Cheboygan and Ocqueoc Rivers, Michigan) and another into Lake Michigan (Carp Lake River, Michigan) when they were intercepted. During their spawning migration, both sea lamprey sexes exhibit the same response to conspecific alarm cue (Bals & Wagner, 2012). However, behavioral response diminishes with sexual maturation in female sea lamprey (Bals & Wagner, 2012), thus we used only males as behavioral respondents. Sea lamprey were transported to HBBS, separated into same‐sex groups via external characteristics (Siefkes et al., 2003) and held in 1,000 L tanks receiving water drawn from Lake Huron (100% exchange every 2 hr). Experimental animals were observed daily for normal activity levels. Only those individuals without sign of external injury or disease were selected for trials, and every individual was used only once. Animal handling and experimentation protocols were approved by Michigan State University Institutional Animal Care and Use Committee, and conducted under the permit AUF 01/14‐007‐00.

### 2.4 Experimental trials

At approximately 1700 hr each day of trials, five groups of sexually immature male sea lamprey (10 individuals per group) were held in cages within the upstream portion of each raceway. At 2200 hr, a trial commenced when a holding cage was moved downstream into the center of the experimental arena of a raceway and sea lamprey released from the cage. Each trial lasted 30 min in total, consisting of a 10‐min prestimulus period and a 20‐min stimulus period. The prestimulus period started when six or more sea lamprey were seen to be exploring the experimental arena. During a stimulus period, one of eight stimuli, or a solvent control, was pumped into one side of each raceway. Ten replicates of each odor were conducted. To control for any effect of raceway identity or odor application side, stimuli were pumped into raceways on alternate sides as well as alternated across replicates within each raceway. After each trial concluded, the total length (mm) and wet weight (g) of each individual sea lamprey used were recorded.

### 2.5 Data analysis

The position of an individual sea lamprey's head was determined every 30 s from the beginning of each trial. An animal's position was assigned to the stimulus (ethanol control or alarm cue application side) or nonstimulus side of either raceway. To allow for the distribution of sea lamprey to stabilize following introduction of stimulus odors, only the final 10 min of the stimulus period was used in data analysis. The proportion of animals positioned on the stimulus side was then calculated for each trial and treatment means plotted. Proportional data were arcsine‐transformed prior to statistical analysis. To confirm there was no effect of raceway identity or side of the raceway receiving a stimulus odor on the distribution of sea lamprey during the stimulus period, a generalized linear model (GLM) containing “stimulus identity” (extracts from American brook, chestnut, northern brook, Pacific, sea and silver lampreys, Atlantic hagfish and white sucker, and ethanol), “raceway identity,” and “application side” as fixed effects was conducted. “Stimulus identity” was found to have a statistical effect (*F*_8,81_ = 9.7, *p *<* *.001) on the distribution of animals, whereas there was no effect of either “raceway identity” (*F*_1,89_ = 0.49, *p *=* *.508) or “application side” (*F*_1,89_ = 0.27, *p *=* *.142). Therefore, “raceway identity” and “application side” were both excluded from further consideration.

A subsequent GLM was used to determine whether the distribution of animals during trials resulted from behavioral responses to either alarm cue odors or an ethanol control. Arcsine‐transformed proportions of sea lamprey on the stimulus side were held as the response variable, while “stimulus identity” was a fixed factor, and water temperature and discharge were used as covariates. Dunnett's *t* tests were conducted post hoc and used to identify statistically significant differences in the proportion of animals on the stimulus side in the presence of alarm cue extracts versus the ethanol control. Lastly, the relationship between the proportions of animals on the stimulus side and divergence times between all odor‐extracted species and sea lamprey was resolved using linear regression. Divergence times were derived from TimeTree (Kumar, Stetcher, Suleski, & Hedges, 2017) and Bartels, Docker, Fazekas, and Potter (2012). All statistical analyses were performed in IBM SPSS Statistics (v. 24) at α = 0.05 and graphed using SigmaPlot (v. 12).

## 3 RESULTS

**Figure 2:**
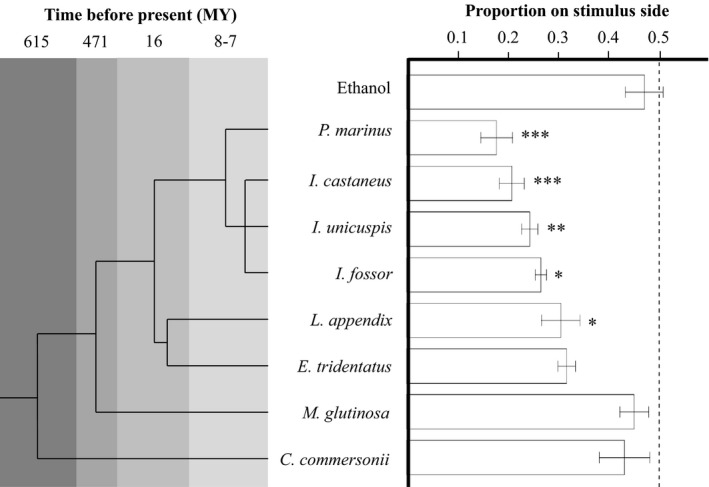
Phylogeny of species whose chemical cues was extracted in this study and behavioral response of sea lamprey (*Petromyzon marinus*) to those odors. The chronogram (left) indicates estimated time of divergence (derived from TimeTree and Bartels et al., 2012) between the common ancestor of sea lamprey, chestnut (*Ichthyomyzon castaneus*), silver (*I. unicuspis*), northern brook (*I. fossor*), American brook (*Lethenteron appendix*), and Pacific lampreys (*Entosphenus tridentatus*); sympatric white sucker (*Catostomus commersonii*) that share predators, allopatric Atlantic hagfish (*Myxine glutinosa*) that do not, as well as an ethanol control. Ages are estimated at millions of years before present (MY). The mean proportion (±*SE*) of sea lamprey detected on the stimulus side is shown on the right. Responses marked with an asterisk indicate a statistically significant difference from an ethanol control (*p < .05, **p < .01, ***p < .001; GLM with Dunnett's *t* test, α = .05). *N* = 10 for each bar. The observed proportions are shown, but analysis was conducted on arcsine‐transformed data

A univariate GLM indicated there were statistically significant differences in the distribution of sea lamprey on the stimulus side between treatment groups (*F*_10,89_ = 8.8, *p *<* *.001). These differences were due to “stimulus identity” (*F*_8,89_ = 10.8, *p *<* *.001), and not either “water temperature” (*F*_1,89_ = 0.66, *p *=* *.42) nor “discharge” (*F*_1,89_ = 0.541, *p *=* *.564). Relative to an ethanol control, the odor extracts of sea and chestnut lampreys (Dunnett's *t* test, *p *<* *.001), silver lamprey (*p *<* *.01), and northern and American brook lampreys (*p *<* *.05) altered spatial use in the raceway, such that statistically significantly lower proportions of sea lamprey respondents were positioned on the stimulus side in the presence of those odors (Figure 2). Extracts from Pacific lamprey (Dunnett's *t* test, *p *=* *.151), white sucker (*p *=* *.983), and Atlantic hagfish (*p *=* *1) did not have a statistically significant effect on the distribution of sea lamprey in raceways, relative to the ethanol control.

Sea lamprey responded most strongly to conspecific extracted odor, with the lowest proportions of animals recorded on the stimulus side among treatments (mean ± *SE*, 0.18 ± 0.03, range 0.06–0.35). Chestnut (mean ± *SE*, 0.21 ± 0.02, range 0.1–0.37), silver (mean ± *SE*, 0.24 ± 0.02, range 0.14–0.33), and northern brook lampreys (mean ± *SE*, 0.27 ± 0.01, range 0.21–0.33) elicited a clear, but weaker, response from sea lamprey respondents. American brook (mean ± *SE*, 0.31 ± 0.04, range 0.06–0.49) and Pacific lampreys (mean ± *SE*, 0.32 ± 0.02, range 0.17–0.37) provoked a weaker still response, but which was very similar in magnitude between both species and lower than out‐groups. The extracted odors of white sucker (mean ± *SE*, 0.43 ± 0.05, range 0.19–0.76) and Atlantic hagfish (mean ± *SE*, 0.45 ± 0.03, range 0.31–0.57) provoked little to no behavioral response from sea lamprey, as indicated by the proportion of sea lamprey on the stimulus side in the presence of those odors. The magnitude of responses to these latter two odors appeared to be as repellent as an ethanol control (mean ± *SE*, 0.47 ± 0.04, range 0.3–0.68).

**Figure 3:**
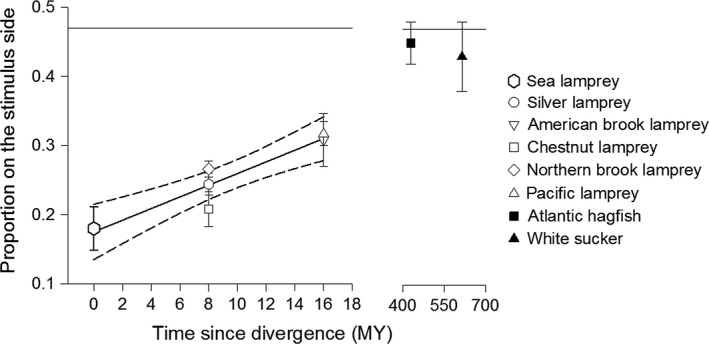
Relationship between the behavioral response of sea lamprey (*Petromyzon marinus*) and the estimated divergence time between the common ancestor of sea lamprey and odor‐extracted species. Shapes represent the untransformed mean (±*SE*) proportion of sea lamprey detected on the stimulus side when exposed to chemical cues derived from members of the family Petromyzontidae (hollow); Atlantic hagfish (*Myxine glutinosa*) and white sucker (*Catostomus commersonii*) (filled). The reference line on the *y*‐axis indicates the mean response of sea lamprey to an ethanol control

Estimated divergence times between taxa used in this study are summarized in Table 1. A statistically significant relationship was found between the mean proportion of sea lamprey positioned on the stimulus side and how distantly related sea lamprey (*Petromyzon* spp.) are from other odor‐extracted species (Figure 3). This relationship explained 80.6% of the variance between stimulus odors (*R* ^2^ = 0.806, *F*_1,6_ = 24.96, *p *=* *.002). As phylogenetic distance increased between sea lamprey and heterospecifics, the strength of avoidance of those extracted odors weakened.

**Table 1:** Estimates of divergence times (MY) and credibility intervals between lampreys (Petromyzontiformes, *Petromyzon marinus*), gnathostomes (Catostomidae, *Catostomus commersonii*), and hagfishes (Myxiniformes, *Myxine glutinosa*). Also shown are divergence estimates between sea lamprey (*Petromyzon* spp.) and silver, chestnut and northern brook lampreys (*Ichthyomyzon* spp.), American brook lamprey (*Lethenteron* spp.), and Pacific lamprey (*Entosphenus* spp.). Estimates are derived from TimeTree based on multiple molecular studies as indicated

Divergence event	Divergence time		Number of molecular studies
	Estimate	Credible interval	
Petromyzontiformes vs Catostomidae	615	524–706	7^a^, ^b^, ^c^, ^d^, ^e^, ^f^, ^g^
Petromyzontiformes vs Myxiniformes	471	391–550	4^h^^,^ i
*Petromyzon* vs *Lethenteron*	16	11–21	1^i^
*Petromyzon* vs *Entosphenus*	16	11–21	1^i^
*Petromyzon* vs *Ichthyomyzon*	7–8	n/a	1^j^

a Wray, Levinton, and Shapiro (1996).

b Otsuka and Sugaya (2003).

c Blair and Hedges (2005).

d Roelents et al. (2010).

e Chen, Zou, Yang, and He (2012).

f Licht et al. (2012).

g dos Reis et al. (2015).

h Hedges (2001).

i Kuraku and Kuratani (2006).

j Bartels et al. (2012).

## 4 DISCUSSION

The strength of response to damage‐released alarm cues declined in close association with increasing phylogenetic distance between sea lamprey and other lamprey species from which alarm cues were derived. By testing alarm cues of representatives from 50% of Petromyzontidae genera, including the most basal sister group (*Ichthyomyzon–Petromyzon*, Gill, Renaud, Chapleau, Mayden, & Potter, 2003), our results suggest that the components of the damage‐released alarm cue in sea lamprey are at least partially conserved within the northern hemisphere lampreys. Notably, American brook lamprey and Pacific lamprey are equally distantly related to sea lamprey (diverged 16 MY) yet elicited a similar magnitude of alarm response—despite the fact American brook lamprey is sympatric with sea lamprey but Pacific lamprey is not. Previous studies have demonstrated that during their spawning migration, sea lamprey are capable of detecting and responding to the damage‐released alarm cue of dead heterospecifics (Bals & Wagner, 2012; Byford et al., 2016), and our data are consistent with and extend those findings. In addition, this study also represents the first reporting of an alarm response by sea lamprey to the damage‐released alarm cue of nonparasitic lampreys (American and northern brook lampreys), species that remain resident in natal streams and do not undertake extensive movements between larval rearing and adult spawning habitats (Malmqvist, 1980). While sea lamprey in this study responded strongly to the alarm cues of conspecific and heterospecific lampreys by avoiding the odor plume, they did not react to the odor of a distantly related yet sympatric species (white sucker *C. commersonii*), an allopatric species (Atlantic hagfish *M. glutinosa*), nor ethanol control. Our findings support the phylogenetic‐relatedness hypothesis of cue similarity.

Recognition of another species' alarm calls/cues is expected to endow “eavesdroppers” with a substantial fitness advantage if an associated antipredator response reduces the risk of predation or improves the chance of escape (Lima & Dill, 1990; Magrath et al., 2015). Thus, the ability of lampreys to detect heterospecific damage‐released alarm cues would be strongly advantageous in both larval and adult life stages. During the spawning migration, when subadults are confined by the river channel and driven to move upstream by the need to spawn, lampreys risk persistent exposure to a gauntlet of diurnal and nocturnal predators and suffer high rates of mortality as a consequence (Cochran, 1986, 2009; Roffe & Mate, 1984; Sepulveda et al., 2013; Sjöberg, 1985, 1989). However, in the absence of visual predator recognition (e.g., in darkness), lampreys would benefit substantially from recognizing and avoiding areas where predators are targeting lampreys, which could be perceived by the presence of confamilial alarm cues (Friesen & Chivers, 2006; Kicklighter et al., 2007; Wisenden, Vollbrecht, & Brown, 2004). Exposure to the damage‐released cue during their spawning migration in natural settings invokes antipredator behaviors in sea lamprey that are similar to the avoidance of the cue in laboratory assays (Di Rocco, Johnson, Brege, Imre, & Brown, 2016; Hume et al., 2015). Further, they appear capable of modulating their response to the alarm cue in a threat‐sensitive manner during migration, depending on the environmental context where the cue is perceived and its spatial extent (Luhring et al., 2016). This strongly suggests migratory sea lamprey that recognize damage‐released alarm cues released from the victims of predator attack flexibly respond to that information in varying contexts to manage the risk of predation.

Sea lamprey do not home to natal streams in order to reproduce (Moser, Almeida, Kemp, & Sorenson, 2015) and have a tendency to overlap spatially with multiple resident and migratory lamprey species across their extensive geographic distribution (Renaud, 2011). They may even do so in response to a phylogenetically conserved migratory‐pheromone produced by all larval lampreys (Fine et al., 2004). Mature sea lamprey also respond to additional pheromones that guide the spawning migration of heterospecific lampreys (Buchinger et al., 2016). Together, this overlap in cues would contribute to the spawning of several lamprey species in the same area as a consequence of similar habitat preferences (Johnson et al., 2015) and the subsequent deposition of larvae of multiple lamprey species in syntopic rearing habitats (Dawson et al., 2015). Thus, the predators of lamprey larvae are shared as well (Cochran, 2009). Larval sea lamprey are able to detect and respond to damage‐released alarm cues by exhibiting reduced drift rates in the presence of the cue, indicating the cue operates throughout ontogeny (Wagner, Kierczynski, Hume, & Luhring, 2016). Furthermore, there is no evidence of life‐stage‐specific alarm cues in sea lamprey as has been reported in juvenile damselfish (Lönnstedt & McCormick, 2011; Mitchell & McCormick, 2013; but see Horn & Chivers, 2017). Instead, larvae respond to the adult alarm cue (Wagner et al., 2016) and adults respond to the larval alarm cue (Bals & Wagner, 2012). Thus, it appears likely that heterospecific cross‐reactivity in alarm cues is retained during the larval life stage.

Chemical compounds that could function as damage‐released alarm cues in aquatic environments are varied in their structure and source (Acquistapace et al., 2005; Brown, Adrian, Smyth, Leet, & Brennan, 2000; Campbell, Coppard, D'Abreo, & Tudor‐Thomas, 2001; Derby & Aggio, 2011; Døving, Hamdani, Höglund, Kasumyan, & Tuvikene, 2005; Howe & Sheikh, 1975; Kelly, Adrian, & Brown, 2006; Zimmer et al., 2006). There is a paucity of evidence to support the idea that any of these compounds evolved primarily to function as an alarm cue, either to preferentially benefit close kin (Russell, Kelley, Graves, & Magurran, 2004) or to attract secondary predators to disrupt the initial predation event (Chivers, Brown, & Ferrari, 2012). Therefore, the function of these compounds as alarm cues likely evolved secondarily as an honest signal of predation when presented alongside additional chemical compounds (e.g., the odor or predators themselves), producing a chemical mixture that cannot be manipulated to the benefit of predator over prey (Bradbury & Vehrencamp, 2000; Guilford & Dawkins, 1991). Among fishes, chemical compounds functioning as alarm cues may have evolved primarily as antimicrobial and UV protective compounds and contained in epidermal cells (Chivers, Wisenden, et al., 2007; Chivers, Zhao, et al., 2007; Faulkner et al., 2017; Halbgewachs, Marchant, Kusch, & Chivers, 2009). Damage to the epidermis of these fish during an attack would release these chemicals into the environment and become available to receivers. If fish alarm cues contain, even in part, those compounds intimately linked with immune function then shifts in niche use following divergence could result in changes to the chemical structure as a function of exposure to novel immune challenges (Mitchell et al., 2012). However, the rate of change may be slow due to the need to maintain a stable immune system; thus, confamilial species may release similar compounds during predator‐induced epidermal damage. Similar, but different, compounds functioning as a damage‐released alarm cue in heterospecifics could still be detected by receivers but elicit a weaker response compared to the compounds released from conspecifics due to a change in the mixture of chemicals present. This is similar to the “blend hypothesis” of insect sex pheromones (e.g., Baker & Cardé, 1979), whereby the full blend of chemicals acting as a pheromone is more attractive to mates than any individual component alone or in combination. Individual larval sea lamprey odor components, or combinations of them, fail to elicit as strong an attractive response to migrating subadults compared to the full odor (Meckley et al., 2012). If lamprey alarm cues evolved in a similar manner, this could explain the observed decline in strength of aversion by sea lamprey in this study to damage‐released alarm cues between more distantly related species.

The sea lamprey alarm cue response is elicited from tissue throughout the body, yet is stronger when derived from the skin (Bals & Wagner, 2012), suggesting the cue is aggregated there but perhaps synthesized elsewhere. Initially, only members of the fish superorder Ostariophysi were thought to possess a damage‐released alarm cue stored in epidermal club cells (Pfeiffer, 1977). However, club cell homologs that exhibit antipredator responses of a similar nature have now been identified in several non‐Ostariophysi lineages (Chivers et al., 2012; Ferrari et al., 2010), including those lacking homologous club cells entirely (e.g., Salmonidae; Smith, 1992), or during ontogenetic stages where club cells are not present (Carreau‐Green, Mirza, Martínez, & Pyle, 2008). Lamprey skin contains no club cells; instead, it contains skein cells (possibly responsible for structural support), granular cells, and mucus cells (Rodríguez‐Alonso, Megías, Pombal, & Molist, 2017). These groups of epidermal cells are also found in hagfishes and teleosts (Spitzer & Koch, 1998; Rodríguez‐Alonso et al., 2017; D. Fudge, personal communication, 2014). Through ontogeny, the epidermis of lampreys increases in thickness, from the pro‐larval stage to the spawning migration (Lethbridge & Potter, 1980, 1981), and in sea lamprey, specifically, this is principally due to an increase in the number of mucus cell layers (Rodríguez‐Alonso et al., 2017). The mucus cell layers of sea lamprey during the spawning migration consist of three discrete types based on a differentiation process as they migrate from the base to the surface of the epidermis: basal, midepidermal, and superficial (Downing & Novales, 1971a, 1971b, 1971c; Rodríguez‐Alonso et al., 2017). Only superficial mucus cells located in the upper layers of the epidermis contain sialic acid and a variety of other glycoconjugates similar to those of the mucus itself (Rodríguez‐Alonso et al., 2017). These compounds provide protection and defense against pathogens for both teleosts (Esteban, 2012; Sarasquete et al., 2001) and lampreys (Tsutsui, Nakamura, & Watanabe, 2007). Although the adaptive immune systems of gnathostomes and lampreys‐hagfishes were acquired separately during evolution (Rast & Buckley, 2013), they exhibit profound similarities (Docker, 2015). It is possible, therefore, that damage‐released alarm cues in lampreys evolved principally for protection against water‐borne pathogens, as has been suggested for teleosts (Chivers, Wisenden et al., 2007; Chivers, Zhao, et al., 2007).

For those species capable of eavesdropping on public information pertaining to predation, the extensive overlap in ecology of larval and adult lampreys represents an opportunity to gain a selective advantage. A shared immune function of damaged‐released alarm cues among lamprey species may be one mechanism by which the process of drift in these chemical cues has slowed over long periods of evolutionary time. To ensure that sea lamprey do not represent a “special case” among lampreys, future studies should test whether other species exhibit the same relationship in their responses to conspecific and heterospecific alarm cues. Furthermore, if the magnitude of those responses among lampreys is found not to correlate with life‐history strategy (e.g., a strong response is present in non‐migratory lampreys), then this would confirm that ecology alone cannot predict the presence–absence of alarm cues within the Petromyzontidae more broadly.

## CONFLICT OF INTEREST

None declared.

## AUTHOR CONTRIBUTIONS

JBH and MW contributed to conception and design of this experiment, as well as analysis and interpretation of the data. JBH acquired the data. JBH and MW drafted and revised this manuscript prior to submission.
